# Genomic insights into the Iron Age Saka of Boz-Barmak, Kyrgyzstan

**DOI:** 10.1038/s41598-026-62891-8

**Published:** 2026-07-20

**Authors:** Aigerim Rymbekova, Pere Gelabert, Alejandro Llanos-Lizcano, Kaaviya Balakrishnan, Michelle Hämmerle, Sojung Han, Olivia Cheronet, Aida Abdykanova, Keldibek Kasymkulov, Michelle Hrivnyak, Jacqueline T. Eng, Ron Pinhasi, Martin Kuhlwilm

**Affiliations:** 1 Department of Evolutionary Anthropology, University of Vienna, Vienna, Austria; 2 Human Evolution and Archaeological Sciences (HEAS), University of Vienna, Vienna, Austria; 3 Department of Anthropology, American University of Central Asia, Bishkek, Kyrgyz Republic; 4 Jusup Balasagyn Kyrgyz National University, Bishkek, Kyrgyz Republic; 5 Institute for Intercultural and Anthropological Studies, Western Michigan University, Kalamazoo, MI USA; 6 Department of Biological Sciences, Western Michigan University, Kalamazoo, MI USA

**Keywords:** Ancient DNA, Human population history, Central Eurasia, Genetic diversity, Kinship and social organisation, Evolution, Genetics

## Abstract

The nomadic cultures of the Iron Age played an important role in shaping the genetic and cultural landscape of Eurasian populations. Yet despite its key geographical location, the Central Eurasian region remains underrepresented in ancient DNA studies of humans. We address this gap through genomic analysis of 12 individuals from the Boz-Barmak burial site in Kyrgyzstan associated with Saka pastoralists (4th−2nd centuries BCE), 9 of which yielded low-coverage genomes (on average 0.7-fold coverage). Genetic clustering analysis placed these individuals within the genetic variation of ancient and modern Central Eurasian and Siberian populations. We found no evidence of first-degree relatives in a kinship analysis, however a network of second- and third-degree relationships seems to be present. Notably, all male individuals share the same Y-chromosomal haplotype, common in present-day Kyrgyz groups, while mitochondrial DNA showed comparably high diversity, with distinct haplogroups observed across the analysed individuals. These findings are in line with archaeological and ethnographic evidence of patrilocality in Early Iron Age Saka, where male lineages remained stable across generations, while female mobility contributed to genetic diversity. Our study complements our understanding of the interplay between kinship, social organisation and population history in nomadic cultures.

**Supplementary Information:**

The online version contains supplementary material available at https://doi.org/10.1038/s41598-026-62891-8.

## Introduction

In the Eurasian Steppe, a dynamic population history with continental-scale migration events took place, with Central Eurasia playing a crucial role as a crossroads of continents, as demonstrated by the westward expansion of pastoralist groups from the Altai region around 5,000 years ago^1, 2, 3, 4, 5^. These migrations into Europe and South Asia are believed to have spread some of the Indo-European languages, fundamentally reshaping the linguistic and genetic landscape of a large part of Eurasia^1, 2, 3, 4, 5^.

The Iron Age (IA) (ca. 1000 BCE – 500 CE) marks an important period in Eurasian history, when nomadic horse-riding cultures emerged as powerful forces that reshaped the genetic and cultural landscape of populations across the continent. Among these groups, the Saka peoples, closely associated with the Scythians, stood out as one of the most prominent IA cultures. It is believed that Saka were a political union of different tribes united by military discipline and at the core of their society stood the kin, an extended unit composed of several families^6^. These kin groups could hold territories, with land regarded as communal property controlled by kin leaders. Some kins were noble houses. Among nomads, the principle of kin-based inheritance of supreme power was rooted in the idea that authority belonged to the entire ruling lineage^7^. According to the genetic evidence, the Saka people likely originated in the Central Eurasian Steppe and were descendants of Late Bronze Age (BA) steppe herders admixed with hunter-gatherers from neighbouring regions^8, 9, 10, 11^. The differences in the sources and proportions of hunter-gatherer ancestry among the people associated with the Saka culture show that groups of the confederation arose from separate local populations in different places^12, 13^. Notably, in some populations, such as Saka of present-day Kyrgyzstan, Iranian Neolithic ancestry is also present^4^, suggesting interaction with the Bactria-Margiana Archaeological Complex (BMAC), a sophisticated sedentary civilization of farmers in present-day Turkmenistan^14^. While these studies provided insights into the origins of Saka, their genetic substructure and kinship practices remain poorly understood.

Here, we report new genomic data from 12 Early Iron Age Saka individuals of the Boz-Barmak burial ground in present-day Kyrgyzstan. The burial ground was excavated during the construction of the Balykchi-Ak-Olon railroad in 2022 by A. Abdykanova (state permit no. 0040/02 20 (17) for rescue excavations of the Boz-Barmak burial complex in 2022. The Boz-Barmak burials are located in the Boz-Barmak mountains, approximately 4 km southeast of Balykchi town, along the southwestern shore of Issyk-Kul Lake (Fig.S1, S2). The individuals from the burial ground were dated over a period between the 4th-2nd centuries BC^15^, which chronologically fits within the Iron Age and presents a rare opportunity to explore the social structure and kinship practices of Early Iron Age Saka people.

## Results

Following standard ancient DNA procedures, we first performed mitochondrial DNA (mtDNA) capture on sequencing libraries from 12 individuals (Methods). Based on the screening, two individuals were excluded from deeper sequencing due to low coverage (BB.K.4, BB.K.8), indicating poor DNA preservation (Table S1). For the remaining individuals, we aimed to obtain approximately 1-fold genome-wide coverage through shotgun sequencing, resulting in coverage between 0.1-fold and 1.4-fold (Table 1). One individual (BB.K.10.2) with very low coverage (0.03-fold) and substantial contamination (16.7%) was removed from further analyses, leaving 9 individuals. Contamination estimates for other samples range between 1.7% and 5.1%, which falls within the expected range for ancient DNA samples. We find typical patterns of ancient DNA damage (Fig S3), and endogenous DNA estimates ranging from 3% to 67.2%. We estimated the coverage on the autosomes and the sex chromosomes separately, which enables us to assign the sex through their respective ratios. Among the nine individuals included in further analyses, six were determined as females and three as males (Table 1).

**Table 1:** Basic metrics on the quality of the samples and genetic sex determination.

ID	Sample Type	Total reads	Endog.%	Modern cont.%	Auto. cov.	X cov.	Y cov.	Sex
BB.K.1	tooth, I1	91,576,637	67.2	2.5	1.4	1.43	0.01	F
BB.Obj.2	tooth, I1	105,353,653	24.7	2.1	0.6	0.62	0.01	F
BB.K.6	tooth, M3	122,390,978	20.0	1.7	0.6	0.31	0.08	M
BB.K.9	tooth, dm2	169,442,108	3.0	2.0	0.1	0.05	0.01	F
BB.K.10.1	tooth, PM2	102,344,280	23.2	5.1	0.5	0.28	0.07	M
BB.K.10.2 (excl.)	bone, petrous	92,365,334	1.5	16.7	0.03	0.03	0	F
BB.K.11	tooth, M2	85,216,391	66.3	1.7	1.4	1.45	0.01	F
BB.K.12	tooth, PM2	120,774,880	32.9	5.1	0.6	0.62	0.01	F
BB.K.14	tooth, M2	140,856,045	13.2	3.0	0.7	0.35	0.09	M
BB.K.15	tooth, M3	119,966,700	27.7	2.1	0.8	0.62	0.01	F

The BB.K.10.2 individual was excluded from further analyses.

(Endog. = endogenous DNA; Modern cont. = modern contamination; Auto cov. = autosomal coverage; X cov. - X chromosome coverage; Y cov. = Y chromosome coverage)

**Fig. 1:**
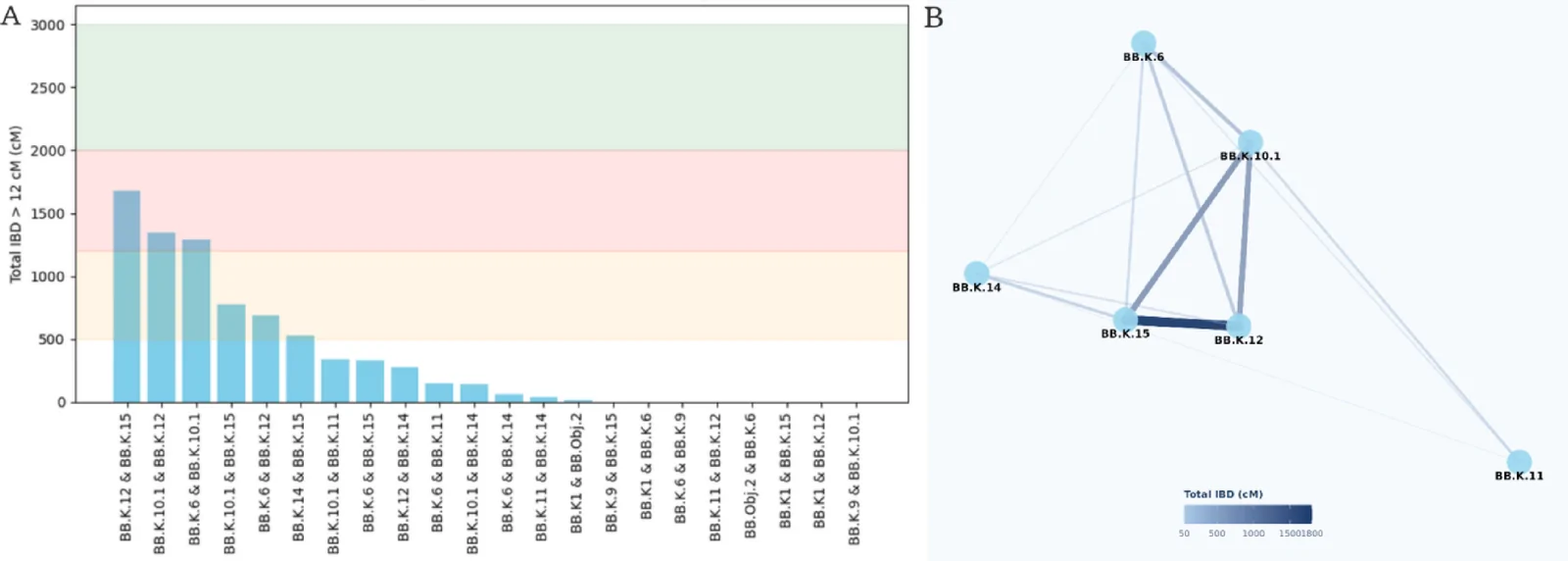
Kinship affinities of the Boz-Barmak individuals within Bronze Age and Iron Age Central Eurasian populations. (A) Pairwise Identity by Descent (IBD) sharing in centimorgans (cM) with relationship threshold ranges: first degree range colored green, second degree - pink, third degree - yellow, higher degree – white. (B) IBD sharing network among the related BozBarmak individuals with nodes representing individuals and edges connecting pairs sharing long (> 12 cM) IBD fragments. Individuals positioned closer share greater amount of IBD. Edge color represents IBD strength: second degree – dark blue, third degree – medium blue, higher degree – light blue.

We explored the biological relationships within our dataset with ancIBD^16^, finding no first-degree relatives. However, we found three pairs of individuals with a second degree of relatedness and three pairs with third degree (Fig. 1A), according to shared IBD fragments longer than 12 centimorgans (cM). Based on the shared IBD fragments, we built a pairwise IBD network (Fig. 1B) to visualise the structure among the related BozBarmak individuals. Only negligible shared IBD fragments were found on the X chromosome. We propose the second degree relationship to be half-siblings, grandparent/grandchild or aunt/uncle/ niece/nephew on the paternal side.

We also explored inbreeding within our dataset using hapROH^17^ by analyzing co-inherited identical haplotypes, or runs of homozygosity (ROH). Most individuals had no detectable ROH, likely indicating absence of close kin unions. Based on the amount of ROH and the number of segments found in three other individuals, we suggest that the parents of those individuals could have been fourth or fifth cousins (Table S2, Fig. S4). We detect low levels of IBD sharing with previously published Bronze Age and Iron Age Central Eurasian individuals, suggesting distant but detectable genetic connections of Boz-Barmak individuals with neighboring groups (Fig. S5).

**Fig. 2:**
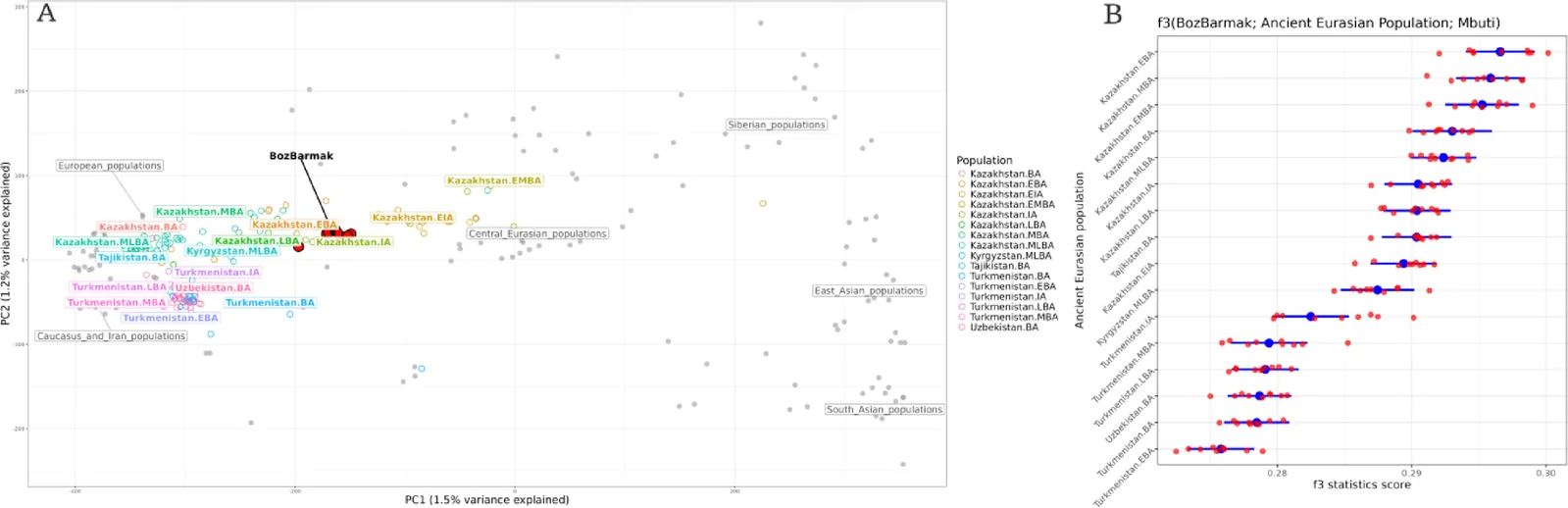
Population affinity of Boz-Barmak individuals. (**A**) Principal component analysis of Bronze Age and Iron Age Central Eurasian populations and Boz-Barmak individuals, based on genome-wide captured data for common human variation, in relation to the modern Eurasian reference panel shown in grey. (BA = Bronze Age, EBA = Early Bronze Age, MBA = Middle Bronze Age, MLBA = Middle Late Bronze Age, LBA = Late Bronze Age, IA = Iron Age, EIA = Early Iron Age). (**B**) The f3-statistics score distribution of Boz-Barmak individuals with Bronze Age and Iron Age Central Eurasian populations. The blue dots represent per population values with standard error intervals and the red dots are per individual values. (BA = Bronze Age, EBA = Early Bronze Age, MBA = Middle Bronze Age, MLBA = Middle Late Bronze Age, LBA = Late Bronze Age, IA = Iron Age, EIA = Early Iron Age).

We performed a Principal Component Analysis (PCA) using BA and IA populations from Central Eurasia, based on data obtained using targeted capture, intersected with genotype calls at the target variants across all nine Boz-Barmak individuals (Fig. 2A). The PCs were computed using modern Eurasian individuals, while the Boz-Barmak individuals together with BA and IA populations were projected onto the top two PCs. The Boz-Barmak individuals exhibited genetic affinity in particular to IA individuals from Kazakhstan. These results were corroborated with outgroup f3 statistics (Fig. 2B): the test f3(Boz-Barmak; Ancient Eurasian population, Mbuti) showed the strongest shared genetic drift between Boz-Barmak individuals and ancient Kazakh populations. While other Boz-Barmak individuals are forming a tight cluster together on the PCA plot, one individual (BB.K.11) stands slightly apart from the rest of the group. The f4 statistics in the form (Mbuti; Population X; one Boz-Barmak individual; all other Boz-Barmak individuals), where Population X is a modern or ancient Eurasian population, further supports this pattern. BB.K.11 is significantly more related to the Siberian/East Asian populations than to the remaining Boz-Barmak individuals, while none of the other individuals showed such strong affinity to any other external population (Fig. S6). Notably, IBD analysis revealed approximately 400 cM of shared segments between BB.K.11 and BB.K.10.1 and about 200 cM between BB.K.11 and BB.K.6, indicative of a relatively close biological relationship (4th−6th degree). This suggests that despite sharing recent kinship with at least these two individuals from the site, BB.K.11 carries a distinct ancestry component that sets this individual apart from the group. Taken together, the results indicate that, with the exception of BB.K.11, the Boz-Barmak individuals have a shared ancestral background with the Iron Age populations of Kazakhstan.

The mtDNA analysis resulted in assigning each individual a different haplogroup, reflecting a mix of East and West Eurasian maternal lineages (e.g. C4a, HV6, K2a, U2e). The male individuals were all assigned to one Y chromosome haplogroup, R-S23592 (R1a1a1b2a2a3), which is commonly found in present-day Kyrgyz individuals (Table 2).

Finally, we screened the sequencing data for genetic traces of potential pathogens. Although we observed matching reads for multiple potentially pathogenic lineages, we did not obtain strong evidence for specific viral, bacterial or fungal infections in these individuals (Table S3).

**Table 2:** Mitochondrial DNA coverage and haplogroup assignment.

ID	mtDNA coverage	Haplogroup
BB.K.1	54	C4a1a3d
BB.Obj.2	46	U4a
BB.K.4	7	U5a1a1
BB.K.6	44	HV6
BB.K8	9	C4a1a
BB.K9	22	U5a1
BB.K10.1	29	U2e2
BB.K10.2	1.5	HV6
BB.K11	88	U4b1a4
BB.K12	46	K2a5b
BB.K14	45	U2e2a1d
BB.K15	50	U5a1a1

## Discussion

In this study, we present genetic data from 12 Early Iron Age pastoralists from the Boz-Barmak burial site in present-day Kyrgyzstan, with 9 low-coverage whole genomes with on average 0.7-fold coverage. The Boz-Barmak individuals were shown not to have any close relatives in previously published individuals from BA and IA across Eurasian Steppe. The genome-wide analysis placed the Boz-Barmak individuals in the broader context of genetic diversity across Eurasian Steppe. However, as a group they demonstrated genetic affinity in particular to IA Kazakh populations.

Beyond broad ancestry, the genetic evidence provided insights into the social structure and kinship practices of Early Iron Age Saka people at Boz-Barmak. The analysis revealed that the burial ground was organized around a network of second-, third- and higher-degree genetic relationships with no evidence of recent parental relatedness. Particularly, one female individual, while sharing IBD fragments with two male individuals indicative of 4th-6th degree biological relatedness, shows a distinct ancestry profile compared to the rest of the group with significant Siberian/East Asian affinities detected via f4 statistics. This co-occurrence of kinship and a notably diverse ancestral background suggests that within this network individuals from varied lineages could have been tied into a single group or extended family. This interpretation is corroborated with the patterns in uniparental markers. The high diversity in mtDNA haplogroups and the predominance of a single Y chromosome lineage found in the Boz-Barmak individuals might reflect patrilocal practices. Findings from other Scythian groups and broader Eurasian steppe populations^2, 18^ have been interpreted to show a similar pattern, leading to the hypothesis that the males may have remained within their natal groups, while females moved between communities. The genetically distinct, yet biologically related female at Boz-Barmak may therefore represent a case of this broad social pattern.

Taken together, our findings support that Boz-Barmak individuals show a particular genetic affinity to Iron Age Kazakh populations within the broader regional population structure of the Eurasian Steppe. The presence of a genetically distinct female who nonetheless shares biological relatedness with male individuals at the site is consistent with patrilocal practices previously documented across the Steppe, where mobile females were potentially introducing diverse ancestries into an otherwise relatively homogeneous kin groups.

## Methods

### DNA extraction and library preparation

DNA extractions and library constructions were performed in laboratories designed and dedicated only to ancient DNA (aDNA) research at the University of Vienna, wearing protective clothing and following aDNA laboratory best practices^19^. Specimens were treated with a sandblaster and ground to bone powder using a MixerMill (Retsch). DNA extraction was performed using an established protocol for aDNA^20^, and single-stranded DNA libraries were prepared^21^. We assessed quantity and quality using an Invitrogen QubitTM 4 Fluorometer and an Agilent 4150 TapeStation. Single-end sequencing was performed on the Illumina NovaSeq 6000 (SP SR100 XP workflow) at the Vienna BioCenter. Mitochondrial capture was performed using myBaits using the manufacturer’s instructions.

### Ancient DNA data processing and quality control

Raw FASTQ data from mitochondrial capture was trimmed with Cutadapt (version 5.1)^22^ and raw reads from shotgun sequencing were trimmed using leehom (version v1.2.18)^23^. All the reads were then mapped to the hg19 human reference genome (GRCh37) http://ftp.1000genomes.ebi.ac.uk/vol1/ftp/technical/reference/ with the Burrows-Wheeler Aligner (BWA) aln algorithm (version 0.7.19-r1273)^24^ using ancient DNA parameters (-t 8 -n 0.01 -o 2 -l 16500). Polymerase chain reaction (PCR) duplicates were removed using Picard MarkDuplicates (version 3.1.1)^25^. Ancient DNA damage patterns were estimated using MapDamage2 (version 2.2.2)^26^ and genotype calling was performed with bcftools mpileup (version 1.22)^27^. Modern human contamination in shotgun data was estimated with Calico (version 0.2)^28^, and endogenous DNA content was calculated as the proportion of uniquely mapped reads (MQ *>* 30) of total sequencing reads. Genotype imputation and phasing of autosomes and chromosome X was performed using the 1000 Genomes Project reference haplotype panel^29^ via GLIMPSE1 (version v2.0.0)^30^. The detailed scripts with parameters used for this study are available here: https://zenodo.org/records/21298537.

## Genetic affinity and kinship analysis

Relatedness between individuals was determined with ancIBD (version 0.7)^16^. ancIBD works with the output from GLIMPSE1 - phased and imputed whole genome vcf files, which are then transformed to HDF5 files. Identical by descent (IBD) segments are called on HDF5 files for each autosome and combined. X chromosome IBD calling was done using ancIBDX command.

Principal Component Analysis for genetic affinities within the region during BA and IA was performed using the smartsnp R package (version 1.2.0)^31^ As the reference panel, EIGENSTRAT files of 153 modern Eurasian individuals and 166 BA and IA individuals from Central Eurasia from the Allen Ancient DNA Resource (AADR)^32^ and Boz-Barmak individuals, filtered for the human 1240k capture panel, using EIGENSOFT package’s (version 8.0.0) convertf and mergeit commands^33^ (346,923 SNPs). The PCs were computed using modern Eurasian individuals, while the Boz-Barmak individuals together with BA and IA populations were projected onto the top two PCs.

f3 statistic analysis was done using the qp3Pop program (version 701), f4 statistics were calculated using qpDstat from the AdmixTools package (version 8.0.2)^34^, implemented through the admixr R package^35^.

Individual ancestry proportions were estimated with ADMIXTURE (version 1.3.0) using the VCF file of Boz-Barmak individuals, filtered for the human 1240k capture panel, converted into PLINK (version 1.9)^36^ binary format (.bed, .bim, .fam). We removed the variants with a minor allele frequency of < 0.01 and pruned the dataset removing all the SNPs in LD with an r^2^ > 0.1, setting a 200-SNP sliding window with a 25-SNP step using PLINK. The pruned dataset consists of 65,375 SNPs. The number of ancestral populations (K) was evaluated across a range from K = 2 to K = 10. The optimal K was determined by running ADMIXTURE with the default 5-fold cross-validation (-cv flag), selecting the K value that yielded the lowest cross-validation error across runs. The final individual ancestry proportions (Q files) were visualised as stacked bar plots.

### Runs of homozygosity

Using a bash script convertVCFtoEigentstrat.sh, the merged VCF file containing Boz-Barmak individuals was converted to the EIGENSTRAT format. Reference haplotype and metadata files for 1000 Genomes reference panel are provided together with the package, as well as Jupyter^37^ notebooks for calling and plotting ROH using hapROH (version 0.64)^17^.

### Haplogroup analysis

Mitochondrial DNA consensus calling was done with bcftools mpileup (version 1.22). Haplogroup assignment was conducted with haploGrouper (version 1.1)^38^. The program takes VCF files of mitochondrial DNA and Y chromosome as input. Pre-computed tree, locus and scores files for haplogroup assignment for both mitochondrial DNA and Y chromosome are provided with the program. Plots were created using custom R scripts (version 4.3.0)^39^.

### Metagenomic assessment

For the metagenomic screening, raw FASTQ files were trimmed using trimmomatic (version 0.39)^40^ and deduplicated with bbmap (version 39.06) clumpify^41^. Taxonomic classification was performed with Kraken2 using a database comprising all sequences from the NCBI nt database. The number of hits corresponding to relevant human-associated pathogens was assessed; however, due to the low number of hits, pathogen screening was not pursued further.

## Supplementary Information

Below is the link to the electronic supplementary material.

Supplementary Material 1

Supplementary Material 2

## Data Availability

Sequence data are available in the European Nucleotide Archive (ENA) under accession number PRJEB97627.
