# Supplementary material for: Genomic insights into the Iron Age Saka of Boz-Barmak, Kyrgyzstan
Source: Sci Rep. 2026 Jul 20;16:33079. doi: 10.1038/s41598-026-62891-8 (PMC13683032; doi:10.1038/s41598-026-62891-8)
Supplement: Supplementary file 1 [file 41598_2026_62891_MOESM2_ESM.docx]

# Supplementary Information

# Burial ground archaeological context

# In 2022, as a result of rescue excavations, the Boz-Barmak burial ground was excavated during the construction of the Balykchi–Ak-Olon railroad. The Boz-Barmak burials are located in the Boz-Barmak mountains, approximately 4 km southeast of Balykchi town, along the southwestern shore of Issyk-Kul Lake (Fig.S1). The burial ground can be dated over a period between the 4th-2nd centuries BC, which chronologically fits within the Iron Age.

# The site consists of 14 single-individual burials aligned in a chain from north to south oriented by head to western sector, though some are positioned outside the main sequence (Fig.S2). The burial mounds have diameters from 7 up to 10–14 meters and reach a height of about 1 meter, indicating that they belonged to the local elite. The burials exhibit a sophisticated construction. The burial pits were covered with timber placed transversely, followed by a layer of shrubs. This structure was then secured and surrounded by clay blocks, which were cut from nearby sources to form the base of the mound. The clay base was reinforced with layers of rock. Additionally, stone rings were placed around the perimeter of the burials to stabilize the mound.

# Analysis of the material culture found in the burials indicates that the burial chain was not organized in a strict north-south orientation but followed a more irregular pattern, probably from center to periphery. Furthermore, our analysis of cultural artifacts and mound construction suggests that the kurgans were built sequentially, from earliest to latest, and may be grouped into three clusters (the following are kurgan numbers): 1) 10.1, 10.2, 8, 5; 2) 11, 12, 13, 14, 15 and object 2; 3) 6, 4, 2, 1 and 3. Kurgan 10 (K10) is proposed as the earliest burial, based on the presence of a rhombus-shaped golden plate decorated with a trefoil motif, as well as a reconstructed handled jar, characteristic of the pre-Hellenistic period.

# Kurgans 5, 8, 10, 11, 12, and 13 are larger in size and appear to be part of the main chain. Kurgans 1, 4, 5, 6, 8, 15 have northern central niches to place pottery set with ritual food. The individual in Kurgan 13 (K13) is interpreted as female, based on the discovery of a gold ring earring with a small soldered ring, similar to findings in kurgans 11 and 10.2, all are females. Later analysis did not yield enough genetic material of the individual in K13 to corroborate archaeological evidence of sex. Kurgans 6, 14 and 15 are smaller in size and appear not to represent leaders of the kin, but rather individuals who played crucial supporting roles in the leadership structure. Kurgan 6 is located outside the main burial chain and has a smaller size. However, it features an outstanding burial mound structure and has been found in situ. In total, only four burials were discovered in situ — Kurgans 4, 6, 13 and object 2. The other burials are presumed to have been looted. Kurgan 6 has two rings supporting the mound and a soft filling in the center. The inner ring was originally a separate mound, composed of rock-covered clay blocks. It closely resembles K13, as both feature two vertically standing rocks in the outer ring, oriented in an east-west direction. The space between these two rocks is enclosed by another rock positioned horizontally. The burial contains unusual artifacts, including an ancient derivative bone buckle adorned with plant motifs, a later-period braid fixator or part of headdress decorated with gold plates, and a small bronze folding mirror with an umbo. Kurgans 4, 2 and object 2 are satellite burials positioned near kurgans 8, 13, 5 and 3: specifically in the northwestern corner with object 2 and in the southern side with kurgan 4 and 2. Kurgan 4 is small in size and is attached to K5 in the south. The individual buried in K4 was young and maybe considered as son and heir of the individual in K5. Kurgan 2 is attached to K3.

# Object 2 is a burial without a mound, consisting of a grave pit with shoulders covered by timber and elongated rocks. The buried woman wore a wooden headdress with rigid outlines, resembling a helmet with holes in the upper and occipital parts. A bronze pin was found vertically positioned in the upper hole of the headdress, and a single bronze earring was discovered next to her left ear. Additionally, the discovery of a horse coccyx bone in the burial, likely as part of ritual food suggests that she held a high social status, signifying respect for her age and position in society.

# Among the burials, only object 2 and kurgans 11, 12, and 13 had grave pits with shoulders. These pits were unusually deep, over 2 meters in the case of kurgans 11, 12 and 13, allowing enough space to lower the deceased inside. The original pit was larger, and at a depth of about 1 meter, another pit was dug in the center, leaving shoulders or ledges around its edges. All the deceased were buried in wooden-frame coffins, possibly covered with felt. Burial pits of K10.2 and K11 contained more elongated coffins, extending up to one meter beyond the head. This extra space may have been used for storing artifacts, which were later probably looted. Despite the looting, the upper part of the woman buried in K11 remained in situ. The preserved shape of a headdress was found on her head, and beneath it, a bronze buckle in the form of a mirror and a bronze pin were discovered. Only women in object 2 and K11 have preserved headdresses. In the mound of K12, scattered remains of the upper body and head were discovered. The primary burial of K12 was found in situ without any material objects.

# Figure S1: The geographic location of the Boz-Barmak burial site in Kyrgyzstan is shown with a golden star. The map was created with MapChart.net. Maps created with MapChart can be freely used, edited and modified for publications, provided that mapchart.net is referenced (<https://mapchart.net/terms.html>, accessed July 13, 2026).

# Figure S2: The schematic representation of the Boz-Barmak burial site created with Canva ([www.canva.com](http://www.canva.com/), accessed July 13, 2026). The site consists of 14 burials aligned in a chain from north (indicated with a black arrow) to south. The map shows a possible chronological sequence of mound construction, based on the current interpretation, with kurgans grouped into three clusters from earliest to latest: green cluster (K10, K8, K5), orange (K11, K12, K13, K14, K15, Object 2) and blue (K6, K4, K3, K2, K1).

# Supplementary Figures and Tables

Table S1: Results from mitochondrial DNA capture (Modern cont. = modern contamination).

| ID | Total reads | Aligned reads | Unique reads | Reads with quality *>* 30 | Modern cont.% |
| --- | --- | --- | --- | --- | --- |
| BB.K.1 | 11,367,543 | 4,583,220 | 33,044 | 32,761 | 2,1 |
| BB.Obj.2 | 12,724,850 | 9,666,832 | 32,932 | 32,220 | 9,9 |
| BB.K.4 | 11,349,329 | 127,446 | 3,350 | 3,123 | 0 |
| BB.K.6 | 11,586,891 | 9,594,249 | 33,072 | 32,553 | 2,7 |
| BB.K.8 | 10,961,268 | 468,843 | 4,181 | 3,922 | 5,1 |
| BB.K.9 | 13,770,508 | 11,805,917 | 33,069 | 32,293 | 1,7 |
| BB.K.10.1 | 11,325,963 | 8,327,901 | 32,906 | 32,175 | 3,3 |
| BB.K.10.2 | 11,286,571 | 4,902,714 | 17,088 | 16,077 | 0 |
| BB.K.11 | 11,100,431 | 7,029,265 | 33,071 | 32,777 | 1,3 |
| BB.K.12 | 13,870,541 | 9,847,270 | 33,068 | 32,007 | 3,1 |
| BB.K.14 | 12,297,707 | 8,741,174 | 33,070 | 32,620 | 2,3 |
| BB.K.15 | 11,676,636 | 7,814,114 | 33,071 | 32,440 | 2 |

Table S2: Total amount of runs of homozygosity (ROH) in centimorgans (cM) and the number of ROH fragments detected

| ID | Sum of ROH (cM) | Number of ROH fragments |
| --- | --- | --- |
| BB.K12 | 31.7 | 2 |
| BB.K15 | 9.5 | 1 |
| BB.K.6 | 6.4 | 1 |

Figure S3: Ancient DNA damage patterns of nine Boz-Barmak individuals.

Figure S4: Runs of homozygosity detected in three Boz-Barmak individuals.

Figure S5: Identity by Descent (IBD) sharing in centimorgans (cM) between Boz-Barmak individuals and previously published populations, including fragments longer than 8 cM. Each point represents an individual of an ancient Central Eurasian population and its shared IBD with Boz Barmak individuals. (BA = Bronze Age, EBA = Early Bronze Age, MBA = Middle Bronze Age, MLBA = Middle Late Bronze Age, LBA = Late Bronze Age, IA = Iron Age, EIA = Early Iron Age)

Figure S6. F4 statistics score distribution of Boz-Barmak individuals with modern and ancient Eurasian populations. Positive values indicate that the tested individual shares more genetic drift with Population X relative to the other Boz-Barmak individuals. Red points show statistically significant results with Z score higher than 3. Error bars represent one standard error.
